# Supplementary material for: Deep analysis of CD4 T cells in the rhesus CNS during SIV infection
Source: PLoS Pathog. 2023 Dec 7;19(12):e1011844. doi: 10.1371/journal.ppat.1011844 (PMC10729971; doi:10.1371/journal.ppat.1011844)
Supplement: S1 Table — (DOCX) [file ppat.1011844.s001.docx]

**S1 Table. Nonhuman primate cohorts.**

| **Group** | **Animal ID** | **Sex** | **Age (years. months) at Nx.** | **Weight (kg) at Nx.** | **Site** | **Virus**  **(1x10^4^ TCID_50_)** | **IgG antibody* control (25mg/kg)** | **ART**  **Regimen** |
| --- | --- | --- | --- | --- | --- | --- | --- | --- |
| Acute 251 Cohort (n=4) | 41812 | F | 11.02 | 7.30 | CNPRC | SIVmac251 | Anti-desipramine | - |
|  | 40742 | M | 12.04 | 12.00 | CNPRC | SIVmac251 | Anti-desipramine | - |
|  | 37164 | F | 16.01 | 10.83 | CNPRC | SIVmac251 | Anti-desipramine | - |
|  | 35488 | F | 17.04 | 10.48 | CNPRC | SIVmac251 | Anti-desipramine | - |
| Chronic 251 Cohort (n=6) | 38889^&^ | F | 14.06 | 11.31 | CNPRC | SIVmac251 | - | FTC/TDF/DTG |
|  | 38919 | F | 14.06 | 8.63 | CNPRC | SIVmac251 | - | FTC/TDF/DTG |
|  | 36056 | F | 18.06 | 9.96 | CNPRC | SIVmac251 | - | FTC/TDF/DTG |
|  | 37274 | F | 16.06 | 8.67 | CNPRC | SIVmac251 | - | FTC/TDF/DTG |
|  | 39359 | M | 14.04 | 13.36 | CNPRC | SIVmac251 | - | FTC/TDF/DTG |
|  | 36511^&^ | F | 17.06 | 10.34 | CNPRC | SIVmac251 | - | FTC/TDF/DTG |
| Control  Cohort 1 (n=4)      Control Cohort 2 (n=12) | 38163 | F | 15.07 | 7.48 | CNPRC | - | - | - |
|  | 40691 | M | 12.07 | 12.18 | CNPRC | - | - | - |
|  | 38691 | F | 14.10 | 13.56 | CNPRC | - | - | - |
|  | 40499 | F | 12.11 | 9.79 | CNPRC | - | - | - |
|  |  |  |  |  |  |  |  |  |
|  | 47161 | M | 3.05 | 6.21 | CNPRC | - | - | - |
|  | 45781 | M | 5.05 | 6.1 | CNPRC | - | - | - |
|  | 46235 | M | 4.06 | 10.58 | CNPRC | - | - | - |
|  | 46551 | M | 4.04 | 7.34 | CNPRC | - | - | - |
|  | 46548 | M | 4.04 | 6.28 | CNPRC | - | - | - |
|  | 47081 | M | 3.06 | 4.3 | CNPRC | - | - | - |
|  | 45721 | F | 5.06 | 9.76 | CNPRC | - | - | - |
|  | 47154 | M | 3.05 | 4.87 | CNPRC | - | - | - |
|  | 46410 | M | 4.05 | 7.76 | CNPRC | - | - | - |
|  | 46354 | M | 4.06 | 8.39 | CNPRC | - | - | - |
|  | 47466 | M | 3.04 | 5.15 | CNPRC | - | - | - |
|  | 47387 | F | 3.04 | 5.05 | CNPRC | - | - | - |

SIV: Simian Immunodeficiency Virus; ART: Anti-Retroviral Therapy; FTC: Emtricitabine; TDF: Tenofovir disoproxil fumarate; DTG: Dolutegravir; CNPRC: California National Primate Research Center

*****Antibody treatments were administered at 1 week prior to SIV infection and every subsequent 10-day interval at doses of 25mg/kg intravenously (neat) over the course of the study.

&, Mamu A*01^+^
