# Supplementary material for: Deep analysis of CD4 T cells in the rhesus CNS during SIV infection
Source: PLoS Pathog. 2023 Dec 7;19(12):e1011844. doi: 10.1371/journal.ppat.1011844 (PMC10729971; doi:10.1371/journal.ppat.1011844)
Supplement: S2 Table — (DOCX) [file ppat.1011844.s002.docx]

**S2 Table. Plasma and CSF vRNA in Chronic 251 cohort and weeks of ART initiation and interruption.**
