# Supplementary material for: Deep analysis of CD4 T cells in the rhesus CNS during SIV infection
Source: PLoS Pathog. 2023 Dec 7;19(12):e1011844. doi: 10.1371/journal.ppat.1011844 (PMC10729971; doi:10.1371/journal.ppat.1011844)
Supplement: S3 Table — (DOCX) [file ppat.1011844.s003.docx]

**S3 Table. Antibody reagents for Flow Cytometry Analysis.**

| **S. No** | **Reagents** | **Source** | **Catalog number** |
| --- | --- | --- | --- |
| 1. | AF700 anti-human CD3 (Clone SP34-2) | BD Biosciences | Cat# 557917 |
| 2. | APC-Cy7 anti-human CD3 (Clone SP34-2) | BD Biosciences | Cat#557757 |
| 3. | BV650 anti-human CD4 (Clone L200) | BD Biosciences | Cat# 563737 |
| 4. | BUV805 anti-human CD8 (Clone SK1) | BD Biosciences | Cat#612889 |
| 5. | BUV737 anti-human CD95 (Clone DX2) | BD Biosciences | Cat# 564710 |
| 6. | PE/Dazzle 594 anti-human CD28 (Clone CD28.2) | BioLegend | Cat# 302942 |
| 7. | APC-Cy7 anti-human CD20 (Clone 2H7) | BioLegend | Cat#302314 |
| 8. | APC-Cy7 anti-human live/dead | invitrogen | Ref#L34976A |
| 9. | PE anti-human CD197 (CCR7) (Clone 3D12) | BD Biosciences | Cat# 561008 |
| 10. | BV785 anti-human CD195 (CCR5) (Clone 3A9) | BD Biosciences | Cat#565001 |
| 11. | PE-CF594 Mouse Anti-Human CD196 (CCR6) (Clone 11A9) | BD Biosciences | Cat# 564816 |
| 12. | BV711 anti-human CD69 (Clone FN50) | BioLegend | Cat#310944 |
| 13. | FITC anti-human CD49d | Beckman Coulter | Part no# IM1404U |
| 14. | PE/Dazzle™ 594 anti-human/mouse Integrin β7 (Clone FIB504) | BioLegend | Cat#321226 |
| 15. | PE anti-human Integrin β1 (Clone TS2/16) | BioLegend | Cat# 303003 |
| 16. | APC anti-human CD183 (CXCR3) (1C6/CXCR3) | BD Biosciences | Cat#550967 |
| 17. | PECy7 anti-human PD1 (Clone EH12.2H8) | BioLegend | Cat# 329918 |
| 18. | FITC anti-human TNF-α (Clone Mab11) | BioLegend | Cat# 502906 |
| 19. | PECy7 anti-human IFNγ (Clone B27) | BioLegend | Cat# 506518 |
| 20. | PE/Dazzle™ 594 anti-human IL-2 (Clone MO1-17H12) | BioLegend | Cat# 500344 |
| 21. | FACS lyse | BD Biosciences | Cat#349202 |
| 22. | FoxP3/ Transcription Factor Staining Buffer set | invitrogen | Cat#00-5523 |
| 23. | Brilliant stain buffer | BD Biosciences | Cat#563794 |

CD: cluster of differentiation; PE: Phycoerythrin; AF: Alexa Fluor; BV: Brilliant violet; BUV: Brilliant ultraviolet; Cy: Cyanine; APC: Allophycocyanin; FITC: Fluorescein Isothiocyanate Conjugate.
