# Supplementary figures and images for: Deep analysis of CD4 T cells in the rhesus CNS during SIV infection
Source: PLoS Pathog. 2023 Dec 7;19(12):e1011844. doi: 10.1371/journal.ppat.1011844 (PMC10729971; doi:10.1371/journal.ppat.1011844)

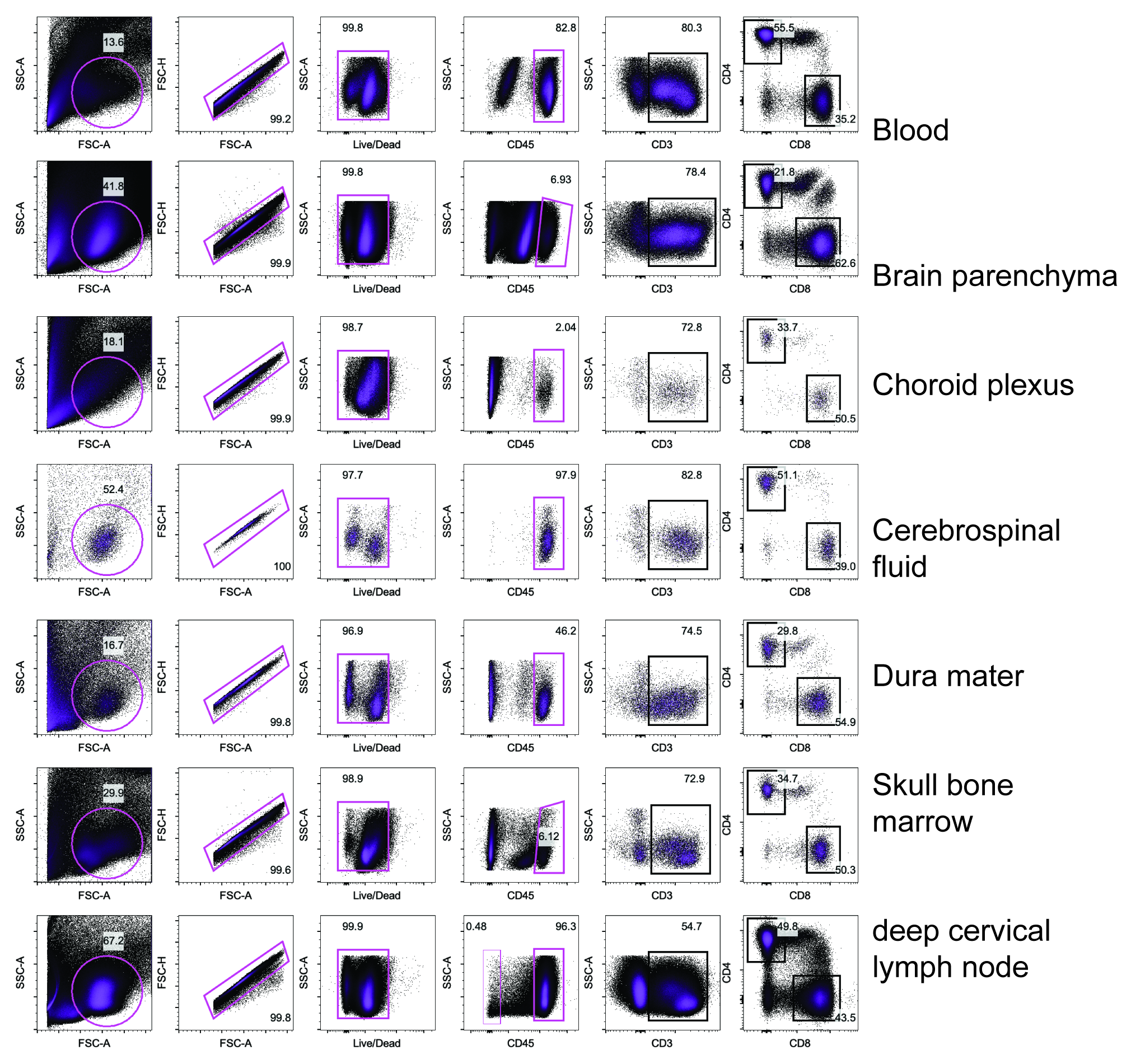

Supplement: S1 Fig — (TIF) [file ppat.1011844.s004.tif]

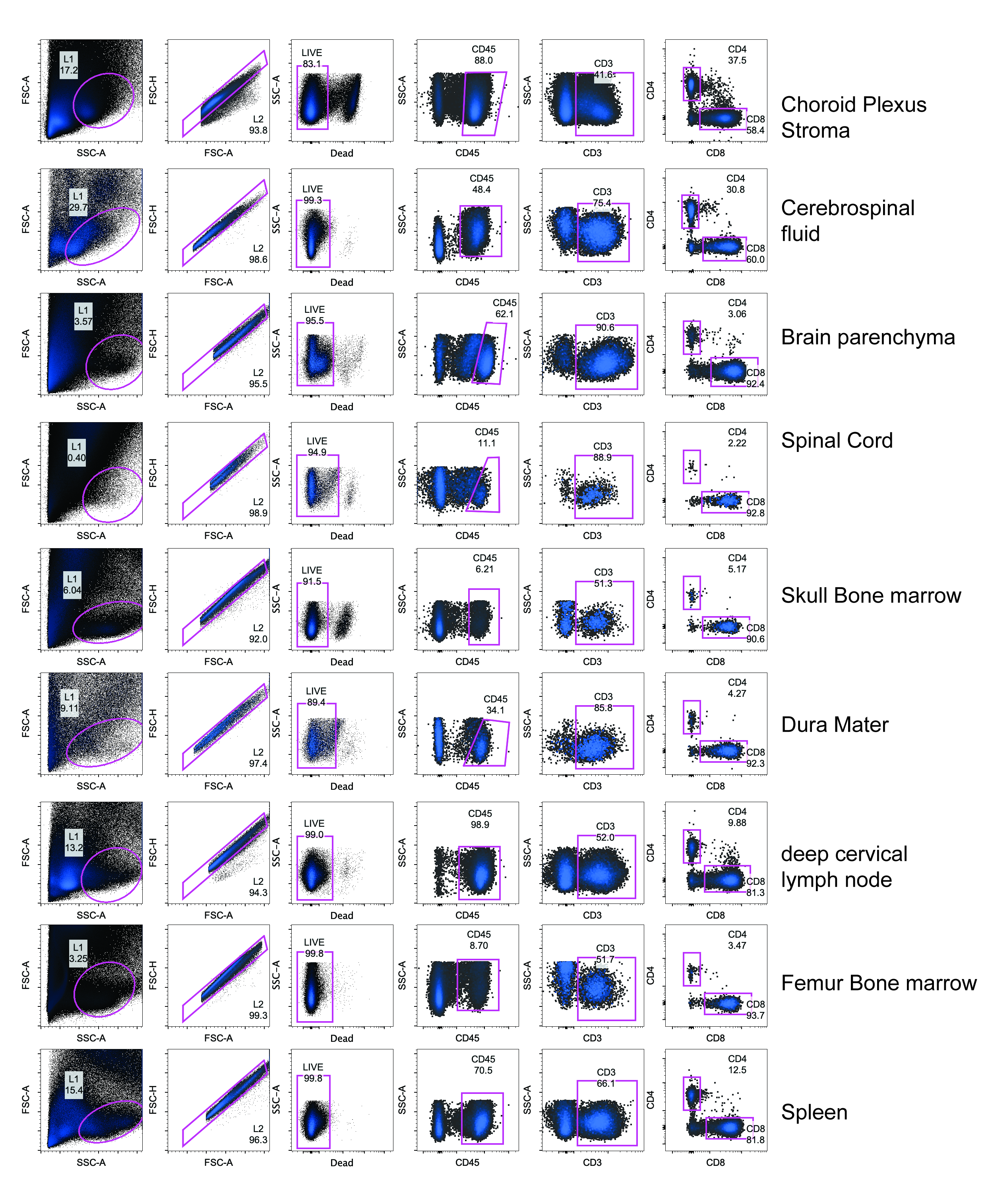

Supplement: S2 Fig — (TIF) [file ppat.1011844.s005.tif]

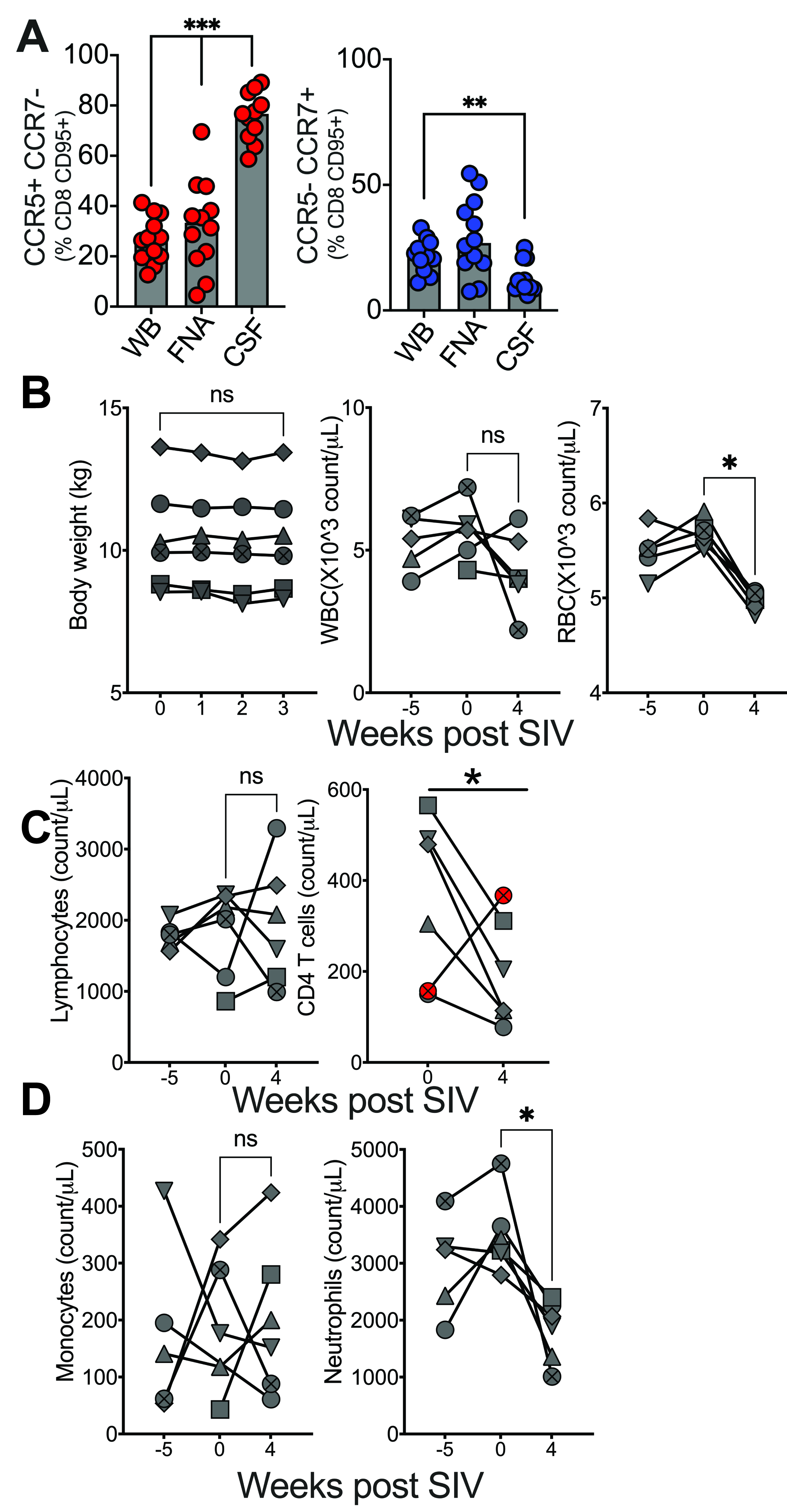

Supplement: S3 Fig — (A) Bar graphs illustrate discrete distribution patterns of CCR5 and CCR7 on CD8+ CD95+ cells in blood, lymph node FNA, and CSF in Control cohort 2 (n = 12). (B) Kinetics of body weight, white blood counts (WBC), red blood counts (RBC). (C) Kinetics of lymphocyte, CD4 T cells, monocyte, and neutrophil counts during first 4 weeks of SIVmac251 infection in Chronic 251 cohort (n = 6). Significant differences by Wilcoxon matched-pairs signed rank test, *, p< 0.05 **, 0< 0.01, ***, p< 0.01. For CD4 T cell counts, p value corresponds to 5/6 animals in gray. (TIF) [file ppat.1011844.s006.tif]

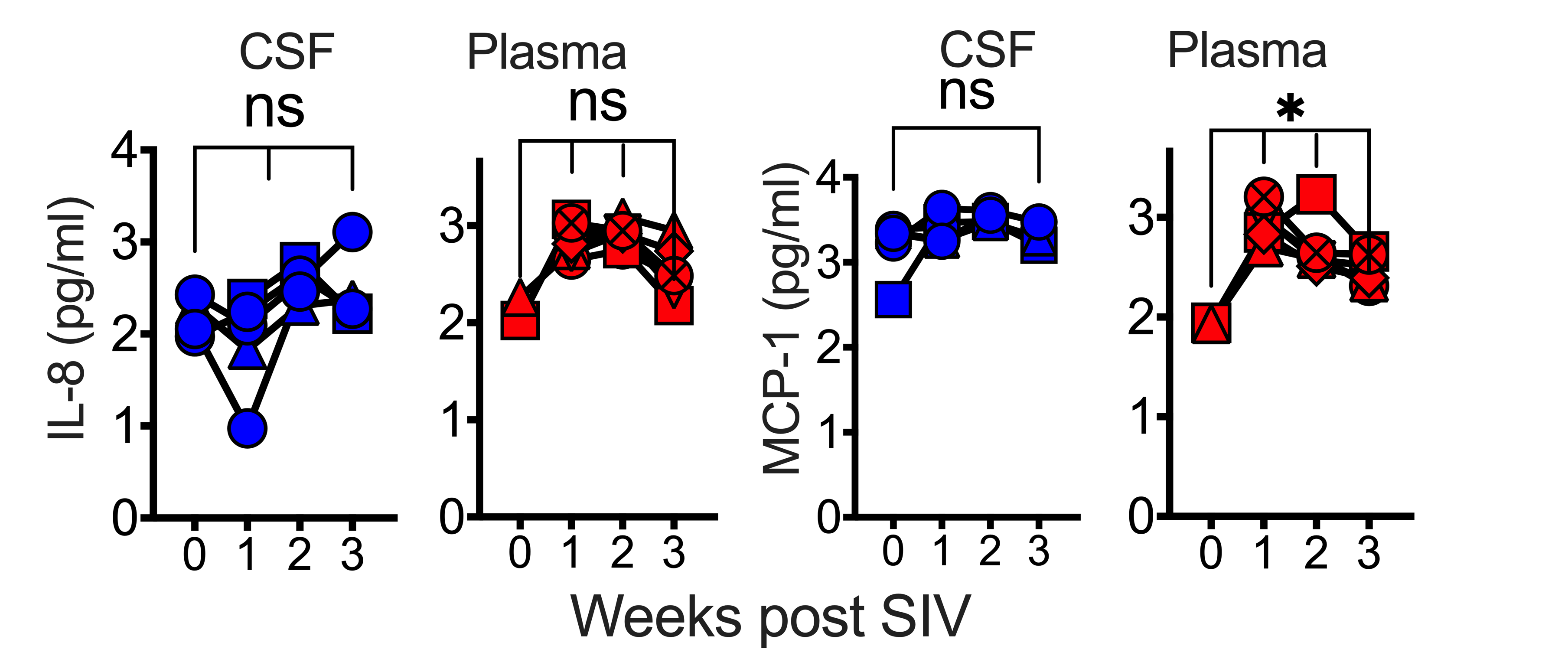

Supplement: S4 Fig — Kinetics of IL-8 and MCP-1 measured by Legend plex assay during first 3 weeks of SIVmac251 infection in Chronic 251 cohort (n = 6). Significant differences by Mann Whitney test *, p< 0.05. (TIFF) [file ppat.1011844.s007.tiff]

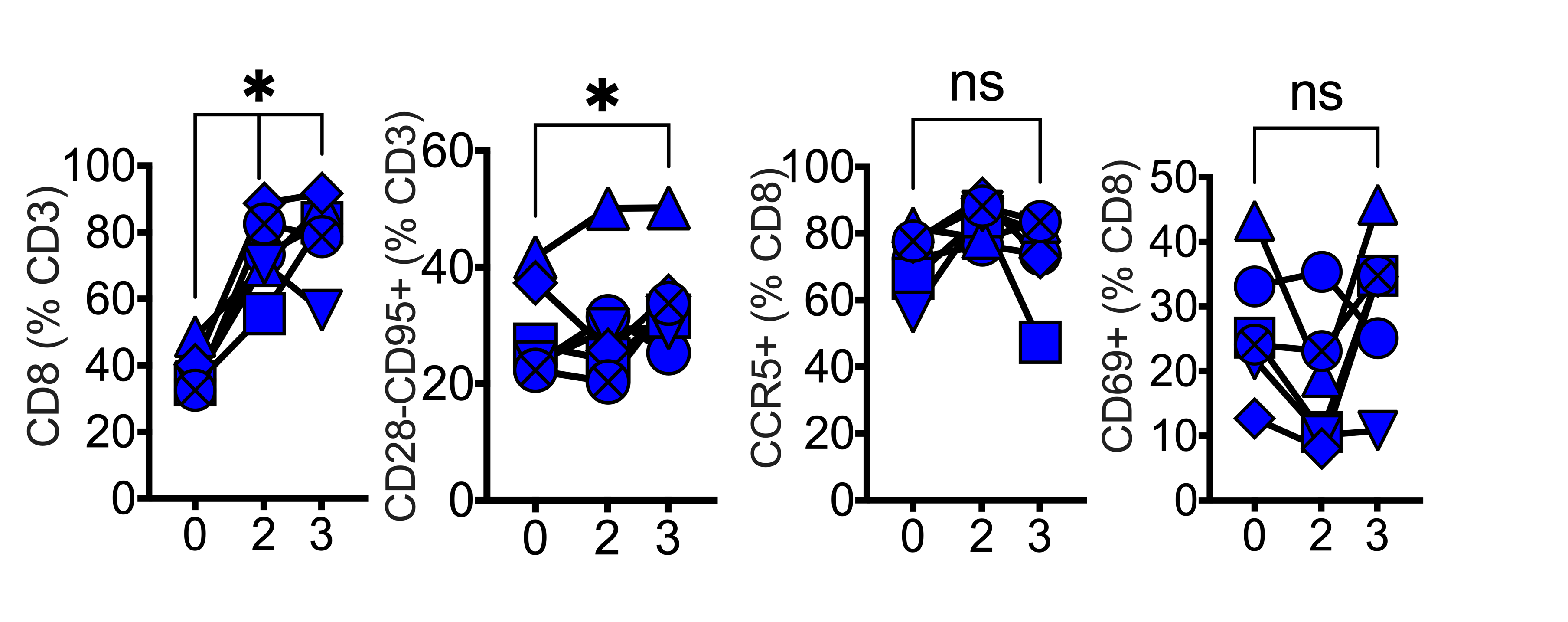

Supplement: S5 Fig — shows % CD8 T cells, % CD28- CD95+ CD8 T cells, %CCR5+ CD8 T cells, and % CD69+ CD8 T cells in CSF in Chronic 251 cohort (n = 6). Significant differences by one-tailed Wilcoxon matched-pairs signed rank test, *, p< 0.05. (TIFF) [file ppat.1011844.s008.tiff]

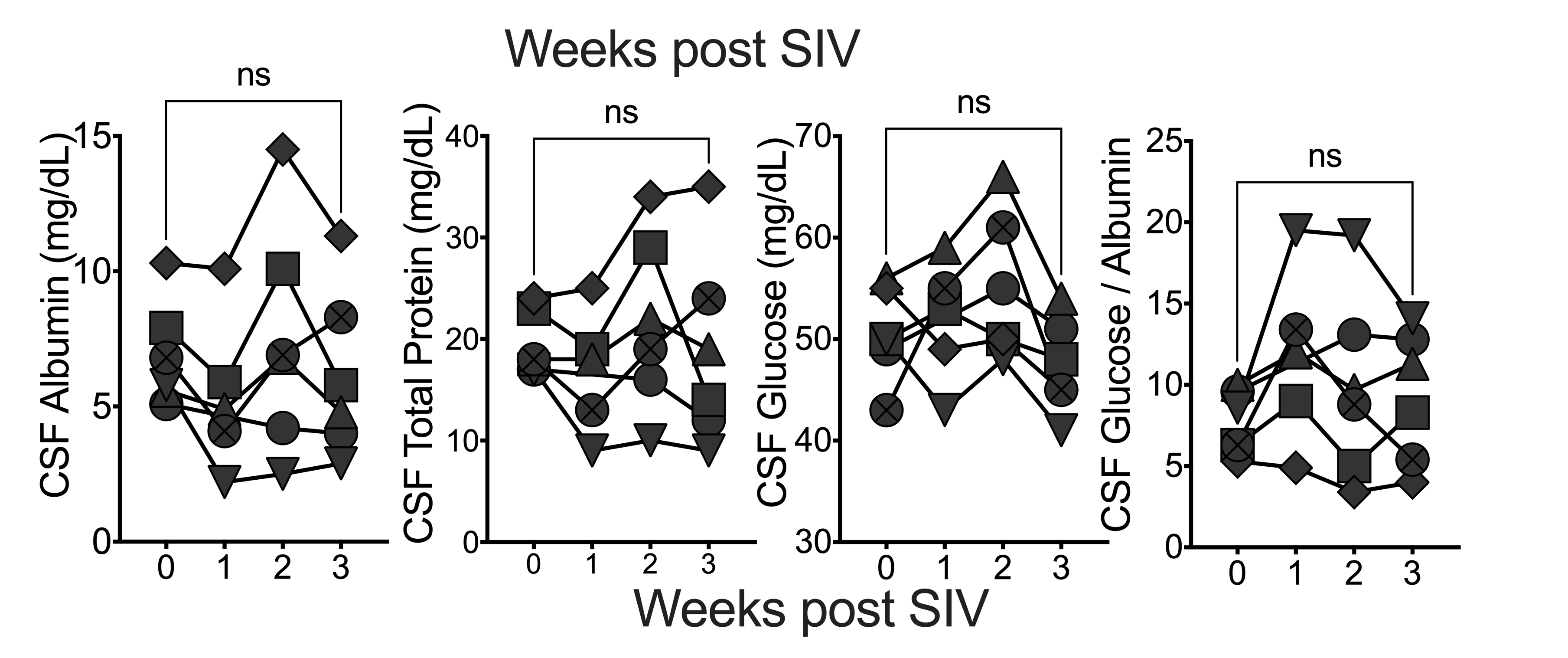

Supplement: S6 Fig — CSF albumin, protein, glucose, and glucose/albumin ratio during first 3 weeks of SIVmac251 infection in Chronic 251 cohort (n = 6). (TIFF) [file ppat.1011844.s009.tiff]

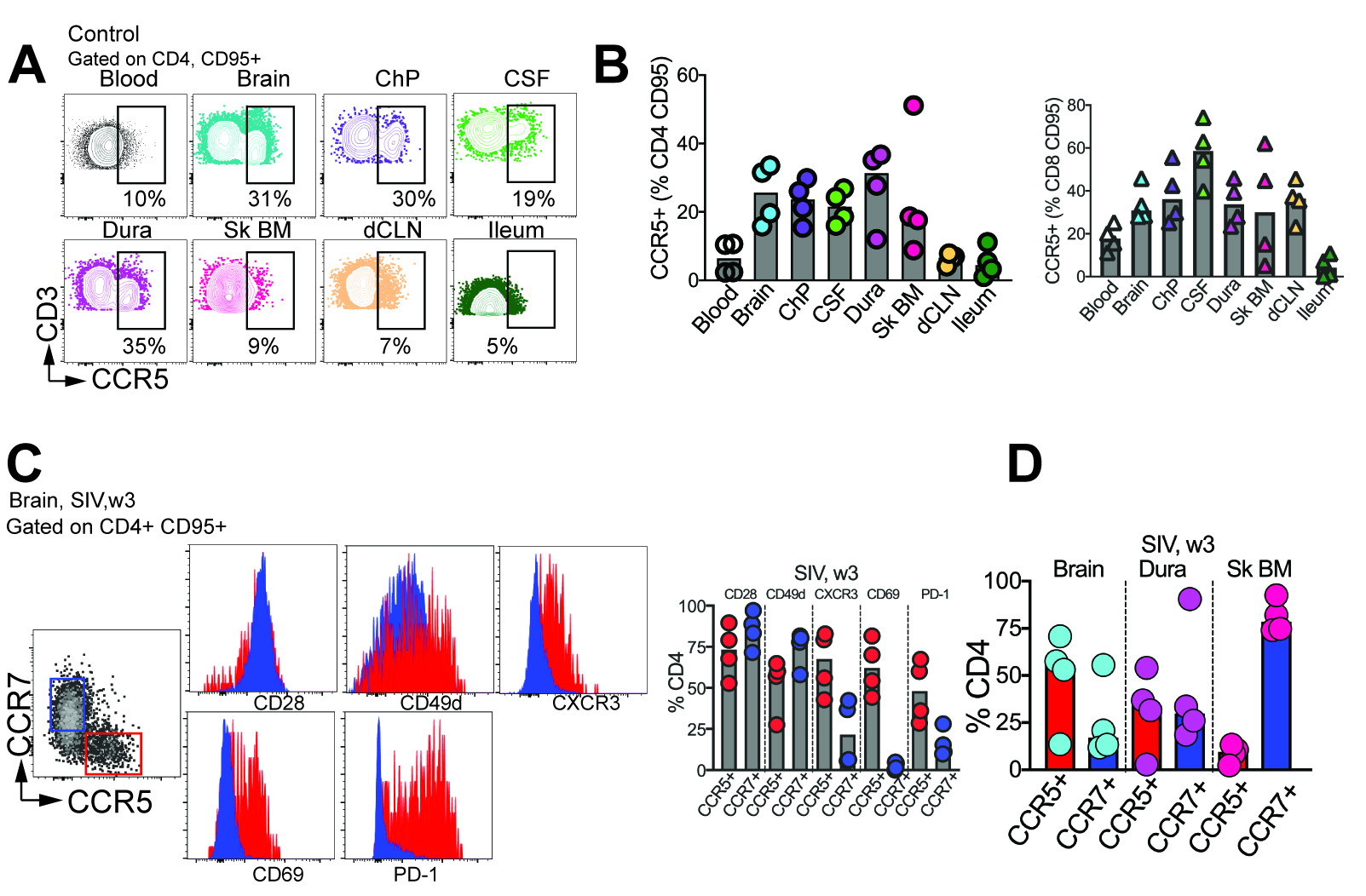

Supplement: S7 Fig — (A) Flow plots and (B) bar graphs show CCR5 expression on CD4+CD95+ T cells in controls, second bar graph shows CCR5 CD8 frequencies. Control 1 cohort (n = 4) assessed. (C) shows phenotype of CCR5+ CCR7- versus CCR7+CCR5- cells. (D) bar graph of CCR5 /CCR7+ CD4 T cell subset frequencies in brain, dura, and skull bone marrow at 3 weeks post SIV. Acute 251 (n = 4) cohort assessed. (TIF) [file ppat.1011844.s010.tif]

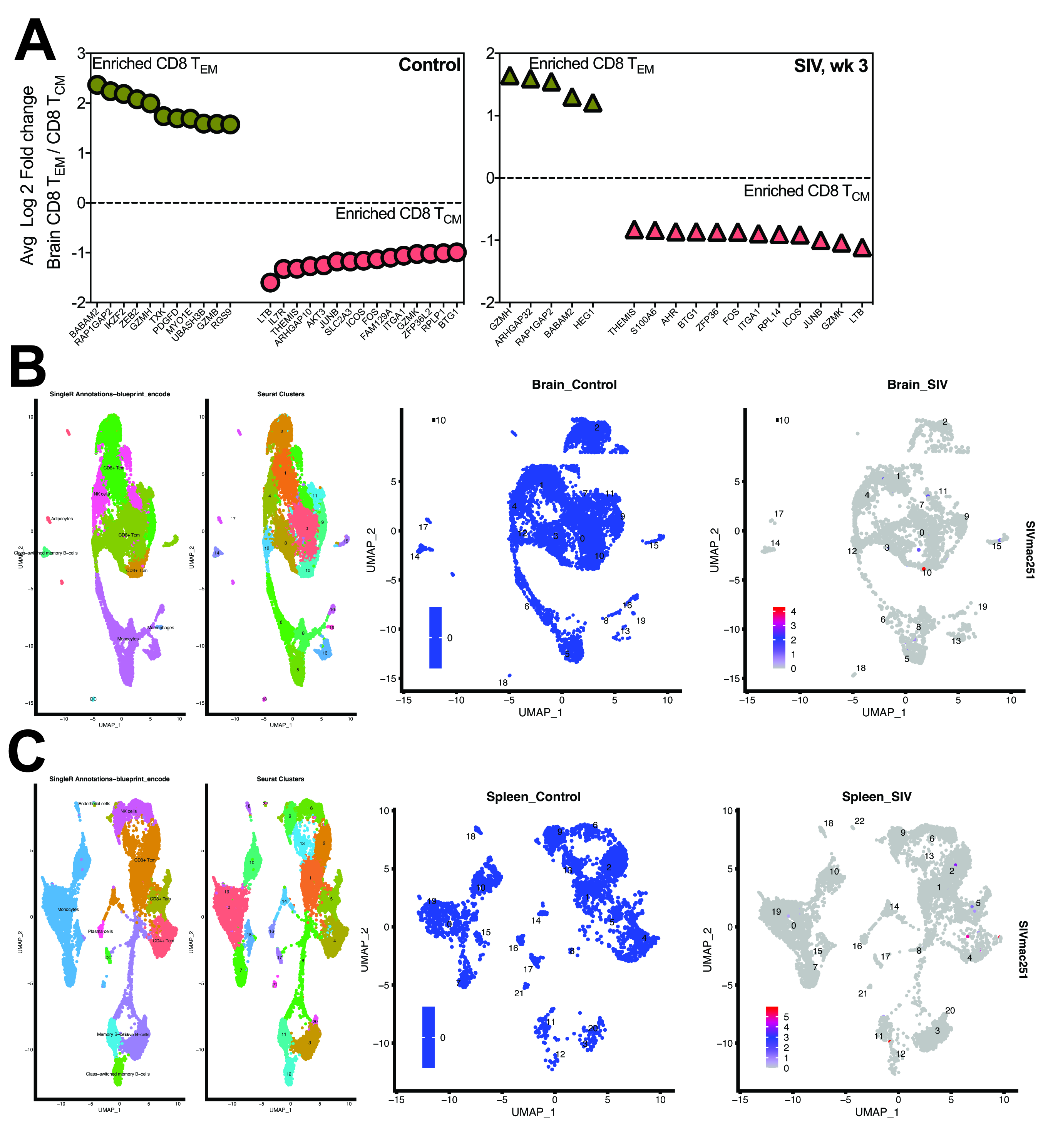

Supplement: S8 Fig — (A) Genes enriched in TEM and TCM clusters in control and SIV brain (p.adj < 0.05). (B) UMAP of immune clusters in brain and vRNA expression in clusters in control and SIV. (C) UMAP of immune clusters in spleen and vRNA expression in clusters in control and SIV. Acute 251 (n = 4) cohort assessed. (TIF) [file ppat.1011844.s011.tif]

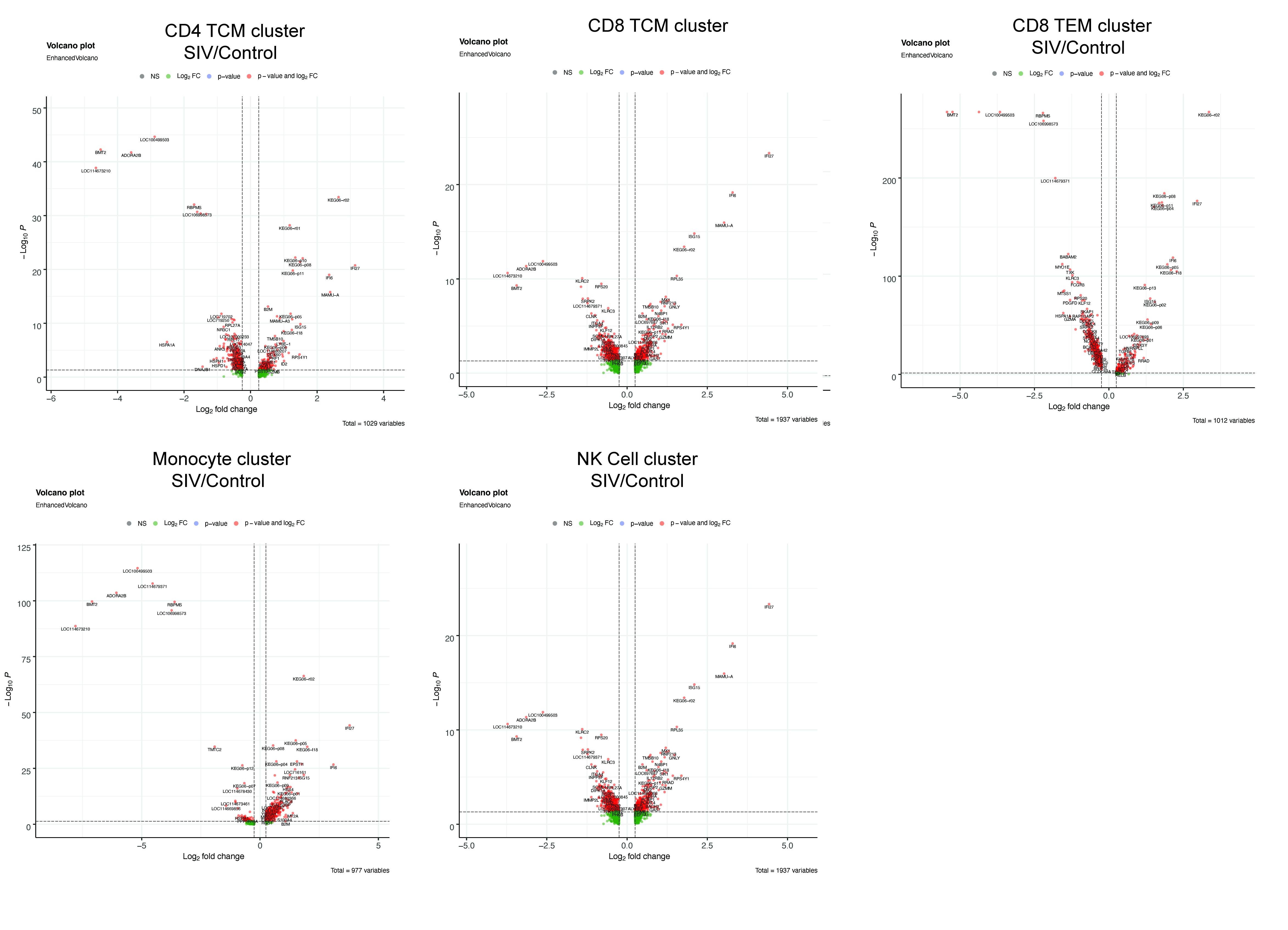

Supplement: S9 Fig — Volcano plots of immune clusters show genes up and downregulated in SIV relative to controls. Genes meeting padj and fold-change cut-off are denoted in red. Acute 251 (n = 4) cohort assessed. (TIF) [file ppat.1011844.s012.tif]

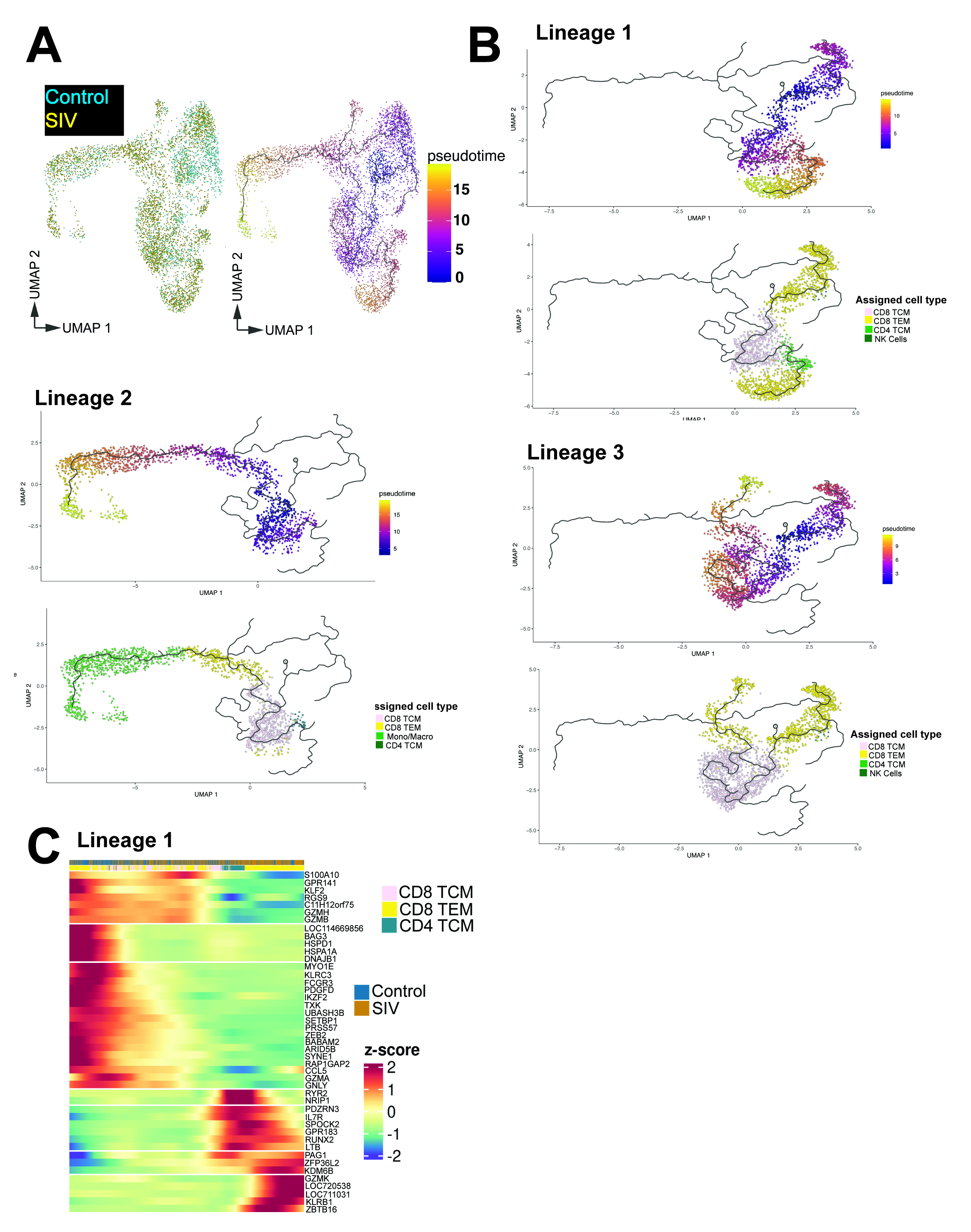

Supplement: S10 Fig — (A) UMAP. (B) pseudotime trajectory comprising of distinct immune clusters shows Lineages 1–3. Lineage 4 comprised only of CD8 TEM. (C) shows heat map comprising of T cell clusters from Lineage 1. Acute 251 (n = 4) cohort assessed. (TIF) [file ppat.1011844.s013.tif]
