# Supplementary material for: Deep analysis of CD4 T cells in the rhesus CNS during SIV infection
Source: PLoS Pathog. 2023 Dec 7;19(12):e1011844. doi: 10.1371/journal.ppat.1011844 (PMC10729971; doi:10.1371/journal.ppat.1011844)
Supplement: Attachment — Submitted filename: Response to reviewers Final.pdf [file ppat.1011844.s015.pdf]

University of Pittsburgh
Division of Experimental Pathology,
Department of Pathology, School of Medicine

Smita S. Iyer, Ph.D.
Associate Professor
Department of Pathology
School of Medicine
Scaife Hall S737
412-624-8890 | siyer.3@pitt.edu

Jason M. Brenchley, Academic Editor
Susan Ross, Section Editor
Kasturi Haldar, Editor-in-Chief
Michael Malim, Editor-in-Chief
PLOS Pathogens

Dear Editors,

We appreciate your coordination of the manuscript review process. We are pleased with the thorough review of our work and acknowledge the valuable comments from the reviewers, which have helped us improve the clarity and quality of our manuscript. In response, we have diligently addressed each of the points raised by the reviewers. Specifically, we have made the following major changes to the manuscript.

1. New data are now presented in Figure 1L-M; Figure 6E-H.
2. We have significantly reworked the Figures and Figure legends to ensure clarity of data presentation.
3. We have included Limitations of the Study section in Discussion.

The manuscript revisions are highlighted, and our responses to each of the reviewer's comments are presented below.

These modifications have strengthened the overall content and presentation of our research. We hope that you will find the revised manuscript suitable for publication in PLOS Pathogens.

Sincerely,

Smita Iyer

Smita Iyer, Ph.D.

Reviewer 1

Concise summary. Part of this study set out to model sub-optimal adherence to ART with 2-3 ATIs and animals were thus viremic for most of the time on treatment. My main concern is that it is unclear how these findings may relate to reservoir or immune abnormalities that persist in fully suppressed settings which is the more clinically pertinent question. The study nevertheless characterizes the CNS in viremic settings with rigor and could be suitable for PLOS Pathogens.

Response. *We acknowledge that our experimental design, focusing on sub-optimal ART, limits our understanding of the CNS in fully suppressive settings. We have now updated the Abstract (**Ln 47-49**) and added a 'Limitations of the Study' section (**Ln 730-742**).*

Major issues. The authors mention SIV-specific responses as data not shown but the authors should really include this and further provide a rigorous characterization or any relation to CNS reservoir. Particularly because (1) the authors show high prevalence of CCR5+ CD8 T cells as they do for CD4s, indicating potential shared trafficking mechanism, (2) transcriptionally active vDNA and thus likely high level of antigen, and (3) CD8 T cell infiltration in multiple CNS tissues in the chronic stages. This has implications for how actively particular CNS tissues are surveilled by viral-specific CTLs, the lack thereof may indicate some degree of sanctuary for SIV persistence.

Response. *We have made significant updates to address these questions. Specifically:*

1. We have now included data demonstrating Gag CM9+ CD8 T cells infiltrating CSF in A*01 animals from our chronic cohort (n=2). Additionally, an inverse association between CSF vRNA and CD69+ CD8 T cells, and increase in CD28- effector memory CD8 T cells in the CSF during acute SIV is presented (***Figure 1L-M, Ln 243-248***).
2. Our primary focus was on CD4 T cells, and we did not perform a rigorous characterization of CTLs in the CNS. However, we have now included phenotypic data suggestive that the brain parenchyma is under surveillance by effector memory (CD28- CD95+) CD8 T cells. During acute SIV infection, an increase in effector memory CD8 T cells in the brain parenchyma is observed. This data is now presented (***Figure 6E-H**, **Ln 470-478***).
3. The question of whether certain cognitively vulnerable regions, such as the PFC and Hp, serve as sanctuary sites is important but is beyond the current study's scope. We have now addressed this limitation in the *Limitations Section* (***Ln 730-742***).

Minor issues

1. The authors may have mislabeled the summary data in Figure 1C. Representative dot plots show 46% CCR5 but the summary data for CSF has a median in the single digits.

Response. We thank the reviewer for bringing this to our attention. We have corrected the labeling of summary data to accurately represent the findings (***now Figure 1B***).

2. If the authors are suggesting rapid influx into the CNS during acute SIV then it would be relevant for the authors to show data on the levels of the R5 ligand MIP-1b in the CSF.

Response. We appreciate the suggestion. However, regrettably, MIP-1β was not included in our measurements.

3. CCR5+ cells appear depleted in CNS tissue, do the target cell densities at these various tissues relate in any way to the CNS or plasma VL?

Response. In response to the reviewer's query, we assessed relationship between target cell densities in CNS tissues and concurrent plasma and CNS viral load. The data show a modest trend for inverse association between target cell densities in brain parenchyma, dura mater, and skull bone marrow and plasma viral load in acute SIV (data presented to the right). We have referenced this association in the text ***(Ln 475-476)***. We did not observe an association between target cell densities and CNS viral load.

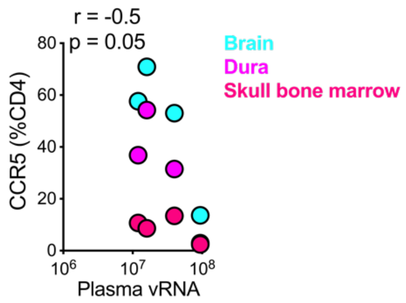

Association between target cell densities in CNS and plasma viral load in *Acute 251 cohort*.

4. Figure 4D. For mapping the SIV transcripts to single cells it would be helpful to provide info on the number and percentage of cells from each cluster that were positive for SIV RNA.

Response. We have now included information regarding the number and percentage of cells from each cluster positive for SIV RNA. This data is now presented in the updated **Figure 4D-E (*Ln 369-384*).** Additionally, we have now expressed the percentage of SIV expression within vRNA+ cells for clarity.

5. Figure 4D, E. For validation of viral read transcripts, It is unclear if a particular threshold was set for read positivity to call a cell positive for SIV RNA, and whether identical thresholds were used for the representative control and SIV sample as the scales appear to be different. As internal controls, the authors should overlay viral read mappings against the entire annotated CD45+ population of an SIV animal as in 4B and not just the Monos/CD4s to show a lack of viral transcripts in refractory populations such as NK or CD8 T cells.

   Response. We appreciate the reviewer's feedback and would like to clarify that identical thresholds (expression value > 1) were consistently applied across all tissues in both control and SIV+ animals (***now stated in Results and legends, Ln 376 -378***). We have now included UMAPs that display the overlay of viral read mappings on the entire annotated CD45+ population of control and SIV+ animals in the brain and spleen. These UMAPs are presented in ***Supplementary Figure 8***.

6. Figure 5A the gene annotations do not align entirely with their corresponding rows and towards the middle of the heatmap particularly it is difficult to determine which gene each row is representative of.

   Response. We have now made the necessary adjustments to ensure that gene annotations align correctly with their corresponding rows in the heatmap in ***Figure 5A***.

7. Figure 7A needs a legend indicating the lines corresponding to plasma or CSF viremia.

   Response. We have included a legend denoting lines corresponding to plasma or CSF vRNA in ***Figure 7A.***

8. While ART was effective at suppressing viremia in the CNS, some of these animals at necropsy still had plasma viremia. This makes for a somewhat heterogenous cohort and it would be informative to denote the data points that correspond to viremic animals in Figure 8 when describing immune abnormalities at the chronic timepoint.

   Response. We have now revised figures (***Figure 8C and 8E***) to distinguish plasma vRNA+ animals from the rest of the cohort.

Reviewer 2

Concise summary. Studies of NeuroHIV in people are limited to analyses of CSF and autopsy samples. Therefore, studies in animal models of HIV/SIV infection in the CNS to understand T cell and virus dynamics are of importance. It is important to note that some of the findings in this group like incomplete recovery of CD4 T cells in the brain and high levels PD1 expression may be due to multiple rounds of ATI in the animals resulting in virus replication and antigen production. The results obtained here may not reflect what occurs in the CNS of people living with HIV that are durably suppressed with ART.

Response. *We appreciate the insightful comments and fully acknowledge the limitations of our experimental design, which primarily focuses on sub-optimal ART. This indeed constrains our ability to draw comprehensive conclusions regarding the CNS in fully suppressive settings. To reflect this, we have now updated the Abstract (**Ln 47-49**) and incorporated a dedicated 'Limitations of the Study' section (**Ln 730-742**) in our manuscript.*

Major issues

1. For the data presented in Figures 1 and 2 and the corresponding supplementary figures, it is not always clear in the manuscript text, figure legends, and figures if the two infection groups (acute and chronic) are being analyzed together or if data from only one group is shown. This should be clear to the reader both in the manuscript text and in the figure legends. In addition, the authors should include the group numbers (n) in each figure legend (all figures).

Response. *To address this comment, we have made several improvements. Firstly, all figures have been revised to clearly indicate the cohorts from which the data are derived. Additionally, we have incorporated the group numbers (n) into each figure legend for both the main and supplementary figures.*

2. For the flow cytometry analysis in Figure 1C and Figure S1 there are ~12 data points. However, there are only 4 animals in the acute group and 6 animals in the chronic group. Are there multiple data points being shown for some of the animals? If so, this should be indicated in the figure legend.

Response. To provide a clearer understanding of data presented, we have taken the following actions. In Figure 1C (***now Figure 1B***), we have clarified in both the figure schema and the legend that the data presented are derived from a separate Control cohort (n=12), which accounts for the larger number of data points. This cohort is now described in the S1 Table.

3. For the single cell transcriptomic analysis, the authors state that the CD8 TCM cluster expressed transcription factors ID2 and JUN and the CD8 TEM cluster expressed the transcription factors ZEB2 and STAT4 and CD69 and Ki67. Based on the presentation of the data in Figure 4C, there does not seem to be much difference in ID2, JUN, or STAT4 expression between CD8 TCM and CD8 TEM cells, especially in the analysis of cells isolated from SIV infected animals. There is also little difference in CD69 expression, if anything it would appear that there is slightly more CD69 expression in CD8 TCM in SIV infected animals.

Response. In response to the reviewer's observation, we have refined our description. We now include genes that are differentially enriched in T_{EM} versus T_{CM} CD8 T cell clusters, providing a more comprehensive account of the transcriptomic differences (***Ln 348-361, S8A Figure***).

4. Figure 5B, C characterizes the genes with upregulated expression in SIV infected animals. Were there any notable genes or pathways with downregulated expression in SIV infected animals? There is mention of downregulated genes in the discussion but not in the results.

Response. To provide a more comprehensive overview of the gene expression changes, we have now incorporated a description of downregulated genes in the manuscript (***Ln 411-419***) and have illustrated in ***Figure 5D***.

5. In Figure 7A and in the figure legend, please indicate which colored line represents the CSF viral load and which represents the plasma viral load. It would appear that blue=CSF and red=plasma. However, if that is the case then it would appear that the first ATI event was initiated in RM 37274 and RM 38359 before the criteria of two consecutive CSF viral loads below 100 copies/ml.

Response. *In Figure 7A, we have now indicated that blue represents CSF viral load and red represents plasma viral load. Additionally, we appreciate the reviewer's observation regarding the exception to the ATI*

*protocol for RM 38359. This was prompted by a decline in plasma viral loads, and we have now included this information in the results section (**Ln 193-195**). ATI was initiated in 37274 at week 10 per criteria. To enhance clarity further, we have included **S2 Table**, which provides plasma and CSF viral loads corresponding to the cycles of ART initiation and interruption.*

6. The manuscript text indicates that RM 37274 was Mamu-A01 positive and had notable CSF viral suppression even without ART but Figure 7a indicates that RM 38889 and RM 36511 were Mamu-A01 positive and it would appear that RM38889 maintained suppression in CSF without ART. Were the labels for RM 37274 and RM 8889 switched? The authors speculate that there was CD8 T cell mediated control in the CSF. Was the virus in the CSF and plasma sequenced to see if there was compartmentalization and/or the emergence of any mutations that would make the virus in CSF less fit?

***Response.** We appreciate the reviewer's keen observation and the opportunity to clarify these points. The manuscript text has been updated to correctly identify the Mamu-A01 positive animal, which is RM 38889, showing notable CSF suppression without ART (**Ln 493**). The colored lines for RM 37274 in Figure 7A (**now Figure 7B**) have now been rectified, with plasma now indicated in red and CSF in blue. As for the sequencing of virus in the CSF and plasma, we must acknowledge that this was not carried out, and we have now explicitly indicated this as a limitation of the study (**Ln 738-742**).*

7. In humanized mice, HIV infection resulted in lower CD4 T cell counts and higher CD8 T cell counts in the brain (PMID: 29863499). Following ART treatment, the CD4 T cell counts in the brain returned to levels similar to uninfected controls. In this study, restoration of CD4 T cell levels was not observed in ART treated macaques. This difference could be due to the fact that animals in this study underwent multiple rounds of ATI and were only on ART for 2-4 weeks prior to necropsy and/or because CD4 T cell levels are reported as a percentage of T cells which is influenced by the actual numbers of CD4+ and CD8+ T cells.

***Response.** We have now referenced the relevant article and provided reasons for the contrasting results in our study (**Ln 546-551**).*

8. Figure 8 shows that significantly more CD4 T cells express PD1 in the brain or dura (either PD1+ or PD1+CD69+) indicating that there is ongoing antigen stimulation in the CSF even with suppressive ART. It is important to note that the animals had multiple rounds of ATI and were only on ART for 2-4 weeks.

***Response.** We appreciate the reviewer's comment and have incorporated it into the manuscript. Specifically, we have now indicated relatively short duration of ART as a contributing factor to the observed immune activation of CD4 T cells in the CNS (**Ln 570-572**).*

<u>Minor issues</u>

1. Figure 1C, it would appear that the labels for FNA and CSF need to be switched in the graph showing %CCR5+CCR7-.

Response. We thank the reviewer for bringing this to our attention. We have corrected the labeling of summary data to accurately represent the findings (***now Figure 1B***).

2. The manuscript text states that CD4 T cell counts in the blood were decreased at week 4. However, there is no graph in Figure S1 or Figure 1 that shows CD4 T cell counts in the blood. Figure 1J shows the CD4/CD8 T cell ratio.

Response. We have updated Figure S1 (***now S3***) to display CD4 T cell counts in the blood, rectifying the absence of this data representation in the previous version.

3. The legend for Figure S1 states that the kinetics of lymphocyte, monocyte, and neutrophil counts is shown for the first 3 weeks of infection. However, the last time point in the graphs is week 4.

Response. We have updated the legend for Figure S1 (***now S3***) to accurately reflect that the counts are shown for the first 4 weeks of infection from Chronic 251 cohort, aligning with the data presented in the graphs.

4. It would be helpful to the reader to have a more detailed explanation of what is being shown in Figure 5E.

Response. We have now expanded the elucidation of the dynamics of ZEB2, GzmB, and LTB within the manuscript (***Ln 433-438***) and included a more detailed Figure legend (now designated as ***Figure 5F***) to provide a deeper understanding of the kinetics of these factors.

Reviewer 3

Concise summary. The strength of the article is the extensive phenotypic and transcriptomic characterization of CD4+ T cells from the brain and surrounding tissues during SIV infection, which make this work novel. It is noteworthy that these tissues were also compared with multiple peripheral tissues. I do have some comments the authors should address to improve the clarify and significance of the manuscript.
Response*. We appreciate the reviewer's summary of our article's strengths. We value the comments and have addressed points raised to enhance the manuscript's clarity and significance.*

Major issues

1. The authors evaluated the functionality of CD4+ T cells after PMA stimulation in the uninfected cohort (Figure 2H). It would be informative if the authors also evaluated the functionality of CD4+ T cells in the acute and chronic cohorts.

Response. We appreciate the suggestion from the reviewer. We conducted an analysis of cytokine production in animals from both our acute and chronic cohorts. While the cytokine production profiles to PMA/Ionomycin appear robust (***figure on right***), due to limitations in sample availability, we are unable to draw rigorous conclusions from these data. Consequently, we have decided not to include these findings in the manuscript.

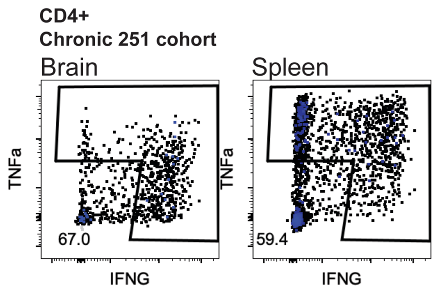

Frequencies of IFNγ, TNFα+ CD4 T cells post PMA/Ionomycin stimulation

2. The authors made multiple statements that there is persistent CD4 depletion in brain parenchyma during chronic infection. It is important to clarify that their study included short ART and multiple cycles of ART interruption, thus it is not designed to determine CD4 reconstitution under prolonged ART. Indeed, in Figure 1E there is a partial reconstitution of total and CCR5+ CD4+ T cells after only 4 weeks of ART. Also, findings related to the viral reservoir presented in Figure 7E and F needs also to be discussed in this context (for example, the sentence . . . underscoring the challenges in eradicating HIV from the CNS, even with effective ART). This is not a model of effective ART.

Response. We acknowledge that it is essential to clarify that our study involved short ART and cycles of interruption. As the reviewer correctly points out, Figure 1E demonstrates partial CD4+ T cell reconstitution after only 4 weeks of ART. We have made the necessary revisions throughout the manuscript and now included a dedicated limitations section (***Ln 730-742***) to emphasize that our model does not represent prolonged ART.

3. 443-447: The authors indicate that there was a rapid decay of CSF viremia on ART up to week 6, suggesting viral output primary due to short lived cells. Then, they added that a second phase of decay was observed in 3 animals, indicating the involvement of long-lived cells as macrophages. First, there is no evidence this is related to macrophage, it can well be longer lived CD4 memory cells. Second, while there are animals in which VL slightly increased on ART, it is difficult to identify 3 animals in which the decay was slower as compared to week 6. Furthermore, all animals but one (week 10) have been on ART only until week 7 or 8. I don't think the study design and the data are supporting that conclusion.

Response. We appreciate the reviewer's insights. Upon further consideration, we have removed the discussion related to the second phase of decay and its potential involvement with long-lived cells, as the study design and data do not strongly support these conclusions.

4. Fig 2H: the authors described the increased levels of IFNγ single-positive cells in the brain, as compared to spleen, but did not mention the lower levels of IL-2+ and TNFa/IL-2 double positive cells, that is the most robust difference between brain and spleen. This needs to be described, and the authors should consider how those differences impact on their sentence "unequivocally demonstrating that Th1 cells in the brain are highly functional (Figure 2H).

Response. We have now revised our description to include the distinctions in IL-2 and TNFα production between brain and spleen. Additionally, we have adjusted our statement to reflect that T_h1 cells in both the brain and spleen demonstrate functionality (***Ln 297-305***).

5. For Figure 4, the authors used RNAscope to confirm the PCR data, revealing vRNA with CD3+ cells in the CNS parenchyma. Viral transcripts were found also in monocyte/macrophage clusters, although at lower level. Does the RNAscope analyses confirm that data as well?

Response. We did observe SIV+ CD3- cells with RNA Scope. To ascertain the identity of these cells, we employed IBA1 as a marker for macrophages. While IBA1 worked effectively in immunohistochemistry (IHC) when used independently, its combination with RNAscope did not yield consistent results. Therefore, based on the morphological characteristics and cellular location, SIV+/CD3- cells likely represent monocytes or macrophages. However, we cannot definitively confirm this observation.

6. Based on the flow cytometry data, it will be interesting if the authors can use their single-cell data to examine transcriptional differences among CCR5+ and CCR7+ CD4+ T cells in the brain of both the unexposed and acute infection cohorts.

Response. We collaborated with our bioinformatician to address this question. Due to the constraints posed by the limited number of cells available in our single-cell analysis (specifically, CCR5 = 16 CD4 T cells in the brain and 7 cells in the spleen, and CCR7 = 47 CD4 T cells in the brain and 91 in the spleen), we are unable to perform downstream analysis to investigate these transcriptional differences.

Minor issues

1. The authors need to work on the flow of the manuscript as it lacks some cohesion. For example, in figure 1 the authors went from describing phenotypically unexposed RMs to describe the kinetics of viral loads and inflammation during acute infection and then to describe the effect of ART. Then in figure 2 they come back to describe the phenotype of unexposed RMs.

Response. *We greatly appreciate the feedback. We have conducted a thorough review and made updates where necessary to enhance the clarity and coherence of the existing content.*

2. Fig 1C is inverted, the graph is showing higher levels of CCR5+CCR7- in FNA than CSF

Response. We thank the reviewer for bringing this to our attention. We have corrected the labeling of summary data to accurately represent the findings (**now Figure 1B**).

3. Line 262: the majority of CXCR3+ cells in the CNS were α4β1+. According to the representative staining, a minority of the CXCR3+ in the CNS are a4b1+, 22%, with large majority of cells being B1+ but a4-. Please clarify. Similarly, line 310: The brain predominantly had CCR5 CD4 T cells; only 12% are reported as CCR5 (single) positive in the representative staining of Fig 3B. The authors should choice an animal more representative.

Response. *We have provided clarification on the relatively higher proportion of $\alpha_4\beta_1$ expression compared to $\alpha_4\beta_7$ and the Boolean analysis employed (**Ln 289-293**). Additionally, we have updated the CCR5 distribution in the brain (**now Figure 3C**) using a more representative plot, as suggested by the reviewer.*

4. 440: Remarkably, 37274 MauA01+ demonstrated notable CSF viral suppression even without ART …. The animal with CSF viral suppression is indicated as 38889 in Fig 7. Please correct accordingly.

Response. *The manuscript text has now been updated to correctly identify the Mamu-A01 positive animal, which is RM 38889, showing notable CSF suppression without ART (**Ln 486**).*

5. The authors should include supplemental figures or files with the complete gating strategies used to generate representative dot plots presented in the figures throughout the manuscript for each tissue.

Response. We have now incorporated supplemental figures, specifically S1 and S2, which provide a comprehensive visual representation of the gating strategy used to identify T cells in CNS tissues for both SIV-unexposed and SIV-infected animals.

Reviewer 4

Concise summary. In this study, the authors are taking a deep look at the dynamic, location, function, susceptibility to infection and phenotype of the CD4+ cell population in the brain and adjacent tissues while comparing with more distal sites and blood. The authors have used numerous cutting-edge assays in a well-designed animal study and have made important reports as the identification of distinct CD4+ subsets.
Response. We thank the reviewer for the recognition of our work.

Major issues

1. It would have been valuable to add the set of data regarding the Gag-specific CD8T cells mentioned on page 9.
Response. We have now included data demonstrating Gag CM9+ CD8 T cells infiltrating CSF in A*01 animals from our chronic cohort (n=2). Additionally, an inverse association between CSF vRNA and CD69+ CD8 T cells and increase in CD28- effector memory CD8 T cells in the CSF during acute SIV is presented (***Figure 1L-M, Ln 243-248***). We have additionally now included phenotypic data suggestive that the brain parenchyma is under surveillance by effector memory (CD28- CD95+) CD8 T cells. During acute SIV infection, an increase in effector memory CD8 T cells in the brain parenchyma is observed. This data is now presented (***Figure 6E-H, Ln 470-478***).

2. In general, across the manuscript the authors are using "brain parenchyma" to refer to brain tissue. It would be more accurate and informative to actually have the author naming the area of the brain used for each analysis.
Response. We have revised the manuscript to specify the area of the brain used for each analysis. In general, we assessed the brain parenchyma for flow cytometric analysis. For the vRNA analysis, specific brain regions were employed.

3. With the comment made by the authors on page 10 regarding the importance of T cells in infection but also of free virion, The absence of cell associated VL in the CSF samples needs to be raised. would you have enough cells in the CSF to run the assay?
Response*. We did attempt to quantify cell-associated virus from CSF cells. Unfortunately, we encountered limitations in obtaining an adequate number of cells from the CSF to perform this assay. This is an important point, and we have now included this information in the manuscript for clarification (**Ln 255-257**).*

4. The authors are referring to the immune cell population in the brain as a "robust immune environment rich in T cells". Unless I missed it out of the many figures and data, there is no absolute count of the leukocytes in the brain done on the naive or infected animals? there is % of each population gated out of CD3+ cells but not an actual absolute count of each immune cell population before and after infection. Without those data it is difficult to judge of the robustness of the immune cell population in such a large tissue.
Response. We appreciate the reviewer's point, and we have adjusted our description of the T cell immune landscape in the brain. The revised text now emphasizes the existence of distinct CD4 and CD8 T cell subsets in the brain (***Ln 278-281***).

5. A few interesting points were not discussed like:
a. The abundance of CD8 T cells in the brain compared to CD4 in SIV negative animals.
Response. We have now emphasized this point by stating the CD8:CD4 ratio of 2.8 in the revised manuscript (***Ln 280-281***).
b. there is no B cells found in the brain of the acute infected RM. Numerous studies have reported infiltration of B cells during inflammation/infection- Did the authors look for B cells?
Response. We did not observe a substantial proportion of CD20+ B cells in the CNS in our analysis.

c. Can the authors give their opinion on the absence of DC in the SIV naive animals.
Response. *We have now stated that the increase in DC cluster in SIV+ animals indicate differential dynamics of this modest immune subset following infection (**Ln 345-346**).*

Minor issues

1. In my opinion the tittle is a little restrictive- the authors are looking at much more than the CD4T cell responses, like localization, phenotype, sensitivity to infection... this is more of a deep analysis of the CD4+T cells in the CNS.

Response. *We appreciate the input regarding the title of the manuscript. To better reflect the comprehensive scope of our analysis, we have revised the title to "**Deep Analysis of CD4+ T Cells in the Rhesus CNS during SIV Infection**."*

2. Figure 1: general observation, the large dots and lines makes the reading of the graph really hard, and I could not tell how many animals were plotted on all those graphs.

Response. *To enhance the clarity of Figure 1, we have reduced the size of the dots on the graphs. This adjustment should make it easier to discern the plotted animals.*

3. Route and dose on graph can be valuable information.

Response. *Route and dose of SIV infection is now included in **Figure 1A**.*

4. gating strategy can be removed to make panel more clear, the bar graph below it is sufficient. is not essential plus the green color for the CSF doesn't match with the blue color used for all other figure in this panel.

Response. *We have streamlined the panel by decreasing size of flow plot, allowing the bar graph to take center stage.*

5. can the authors add the same graph for CD8T cells. Is there any correlation between cell population loss and blood and CSF VL?

Response. We have now included data demonstrating Gag CM9+ CD8 T cells infiltrating CSF in A*01 animals from our chronic cohort (n=2). Additionally, an inverse association between CSF vRNA and CD69+ CD8 T cells and increase in CD28- effector memory CD8 T cells in the CSF during acute SIV is presented (**Figure 1L-M, Ln 243-248**).

6. Figure1S: maybe the weight graph can be replace by CD4 count. Why there is not 10 animals? X axes has 4 weeks as end point was it not 3 weeks?

Response. We have updated Figure S1 (**now S3**) to display CD4 T cell counts in the blood from the chronic 251 cohort (n=6). The data are derived from chronic cohort and hence the sample size of 6. We have now stated that the timepoint was 4 weeks.

7. entire paragraph on page 12 related to CD28 CD95 cells is in my opinion not relevant in this manuscript that has a lot of other data.
Response. We have now removed this paragraph.

8. Can the authors explain how the Choroid plexus was collected for VL? the number of vRNA and vDNA copies seem extremely high for a compartment of the brain that is only and strictly composed of epithelial cells secreting CSF. Was the Choroid plexus pulled out from the hemisphere at different location or was it cut off with surrounding parenchyma which can explain the results obtained by qPCR.
Response. *We have now specified how the choroid plexus stroma (ChP), which contains immune cells such as macrophages and lymphocytes, was specifically distinguished from the brain parenchyma during isolation. As stated in **Ln 268-272**, clean forceps were used to gently extract the ChP from the frontal horn and the body of the lateral ventricle without disrupting the surrounding tissue. The immune-rich nature of the choroid plexus stroma is substantiated by the presence of T cells, illustrated in Figures 2, 3, 8, as well as newly included Figures S1 and S2*

10. The ISH method needs explanation. First the probe used for RNAscope as no information on the ACD website of the part of the genome targeted- the authors mentioned using a GAG probe in the figure legend but their is no other information (sequence targeted and number of Z pairs) in the method or on ACD probe catalogue. Plus, the ref cited is a short communication with no M&M. Why would the authors not use a probe covering a larger part of the genome, especially when looking for rare events in a really large tissue?
Response. The custom-designed probe, rooted in the SIVmac 239 genome, targets gag, pol, and nef genes. Its chief objective is the detection of rare events during the initial stages following SIV inoculations. This probe's design emphasizes sensitivity. We have now included a new reference: Ma, Z.M., Dutra, J., Fritts, L., & Miller, C.J. (2016). Lymphatic Dissemination of Simian Immunodeficiency Virus after Penile Inoculation. Journal of Virology, 90(8), 4093 (***Ln 1065-1066***).

11. The legend of the figure says "shows SIV RNA in parenchyma and perivascular regions of the brain" not sure where we are as there is no other specific marker or phase contrast in this picture.
Response. This description is based on our examination of RNAscope slides. It does not require a specific marker or phase contrast microscope for interpretation.

12. It would have been more significant to have a picture of CD4 and myeloid cells combined to the RNAscope and ideally DNAscope.
Response. We concur that having a combined image of CD4 and myeloid cells in the RNAscope, and ideally incorporating DNAscope, would have added more significance to our study. However, we encountered technical challenges in executing these specific techniques.

13. For Figure 4, the authors used RNAscope to confirm the PCR data, revealing vRNA with CD3+ cells in the CNS parenchyma. Viral transcripts were found also in monocyte/macrophage clusters, although at lower level. Does the RNAscope analyses confirm that data as well?
Response. We found some SIV+/CD3- cells. When we use IBA1 as a maker for macrophage, even though the antibody works well when doing IHC alone, it does not work while combing with RNA scope. According to the morphometry and location, the SIV+/CD3- cells represent monocytes/macrophages.
