# Supplementary material for: Deep analysis of CD4 T cells in the rhesus CNS during SIV infection
Source: PLoS Pathog. 2023 Dec 7;19(12):e1011844. doi: 10.1371/journal.ppat.1011844 (PMC10729971; doi:10.1371/journal.ppat.1011844)
Supplement: Attachment — Submitted filename: Response to reviewers 11-14-23 Final.pdf [file ppat.1011844.s016.pdf]

University of Pittsburgh
Division of Experimental Pathology,
Department of Pathology, School of Medicine

Smita S. Iyer, Ph.D.
Associate Professor
Department of Pathology
School of Medicine
Scaife Hall S737
412-624-8890 | siyer.3@pitt.edu

Jason M. Brenchley, Academic Editor
Susan Ross, Section Editor
Kasturi Haldar, Editor-in-Chief
Michael Malim, Editor-in-Chief
PLOS Pathogens

Dear Editors,

We appreciate the positive feedback of our work. We have addressed all issues raised by Reviewer #2. The manuscript revisions are highlighted, and our responses to reviewer's comments are presented below.

We look forward to seeing our work published in PLOS Pathogens.

Sincerely,

Smita Iyer

Smita Iyer, Ph.D.

Reviewer 1

Concise summary. My comments are adequately addressed, and I have no others. Although the model may bear some resemblance to full virologic suppression, emphasizing the sub-optimal adherence nature of this study is more accurate than relating these findings to long-term persistence and it is important that the authors addressed this. They should be commended for their work.

Response. *We duly acknowledge that our work is focused on sub-optimal ART and cannot be equated to long-term persistence under suppressive ART. We thank the reviewer for commending our work.*

Major issues. None

Minor issues. None

****

Reviewer 2

Concise summary. This manuscript represents a very comprehensive analysis of the T cell compartment in the brain of uninfected and SIV-infected Rhesus Macaques. I thank the authors for addressing the points raised previously and making the requested modifications to the manuscript and figures. I do have additional points that I think should be addressed to improve the clarity of the manuscript.

Response. *We appreciate the recognition of our work. We thank the reviewer for the thorough review of our manuscript and critical suggestions to improve. The additional points provided for improving clarity have been duly considered and addressed.*

Major issues. None

Minor issues.

1. Introduction, page 6, line 138. Please revise to indicate that CD4 T cell depletion in the CNS was not rescued by suboptimal ART.
   Response. *We have changed suppressive to sub-optimal ART on* ***Line 138***

2. Results, page 7, line 163. I believe the text should state that the chronic cohort was followed for 42 weeks not 40 weeks.
   Response. *The reviewer is correct. We have updated 40 to 42 weeks in* ***Line 163***

3. Results, page 12, lines 295-297. Text indicates that the patterns of CCR7 and CCR5 expression on CD4+CD95+ T cells was similar between the CSF and other CNS tissues with the levels of CCR7 being lower in the brain parenchyma. However, in the dura the levels of CCR7 are also lower with more CCR5+ cells compared to CCR7+ cells with the exception of one animal.
   Response. *We have now specified that "However, the frequencies of CCR7+ CD4 T cells were lower in the brain, and on average in the Dura, compared to the CSF" in* ***Line 297****.*

4. Results, page 15, lines 376-378. The text states that the majority of the vRNA-positive cells were concentrated within the CD4 and monocyte clusters. I agree that the majority of the vRNA-positive cells clustered with CD4 T cells but in both the brain and spleen it would appear that there was more signal from the CD8+ T cell clusters (which are not infected with HIV) than the monocyte cluster in Figure S8 B-C. Could the signal in the myeloid and CD8 T cell clusters be background signal?
   Response. *We acknowledge this possibility and have now included a statement to that effect in* ***Line 377****.*

5. Results, page 15 lines 379-384. The text states that in the brain 11 vRNA+ cells were associated with CD4 T cell clusters and 1 vRNA+ cell was associated with monocyte/macrophage clusters and that in the spleen 4 vRNA+ cells were present in the CD4 T cell and monocyte clusters. However, Figure 4D-E indicate that in the brain 14 vRNA+ cells were associated with CD4 T cell clusters and 4 vRNA+ cell was associated with monocyte/macrophage clusters and in the spleen 8 vRNA+ cells were associated with CD4+ T cell and 3 vRNA+ cells were associated with monocytes/macrophages.
   Response. *Addressing the discrepancy on Results, page 15, lines 379-384, we have rectified the inconsistency. We now consistently adhere to a threshold of SIVRNA expression > 1 in both the figure and results.*

6. In Supplementary Figure 10, three lineages are shown in panel B, but the results indicate that there were four distinct lineages identified by the pseudotime approach. Also, what are the two lineages that represent the transition from TCM to TEM in Supplementary Fig. 10B?
   Response. *We have now detailed the lineages in the results, Figure 5E, and Supplementary Figure 10C, along with incorporating this information into the corresponding figure legends.*

7. In regard to IP-10 CSF levels as a biomarker of ongoing viral replication (Results, page 21, lines 533-536), in Supplementary Figure 11, the levels of IP-10 in CSF do vary a little overtime but there doesn't appear to be a consistent pattern where they go down or up during ART treatment.
   Response. *We have now added a statement (**Line 535-537**) highlighting that the absence of a consistent pattern for CSF IP-10 diminishes its potential as a reliable biomarker.*

8. Results, Page 21 lines 539-541. Please make it clear that the chronic experiment reflects the effect of suboptimal ART on CD4+ T cell depletion.
   Response. *We now replaced suppressive with suboptimal ART (**Line 541**).*

9. Much of the text in **Figure 5E** is too small to read. There are some genes that are shown in a larger font but there are other genes mentioned in the manuscript text that are too small to read in the figure.
   Response. *Efforts to universally enlarge the font in Figure 5E were unsuccessful. However, we have specifically enhanced the legibility of genes referenced in the manuscript, such as S100A10, S100A4, and CST7, which were not previously highlighted in larger font within the figure.*

10. The text in **Supplementary Fig 10B** is too small to read.
    Response. *Supplementary Figure 10B has been revised for improved readability.*

11. Results, page 18 line 454. Should the median vRNA copies in the ChP read 5x10^5 and not 0.5x10^5?
    Response. *The values for ChP are indeed 5x10^5(**Line 453**). After a comprehensive review of the Results section, we confirm that all values are accurately presented.*

12. Please carefully check the references to make sure that they are correct. For example, the new text on page 22, lines 546-551 that discusses the results obtained in humanized mice references a paper on HIV infection of tissue resident memory CD4+ T cells.
    Response. *Reference 47 is now rectified. Additionally, all references have undergone a thorough review to ensure accuracy.*

13. For the description of Figure 8 in the results section, line 542 should reference Figure 8 A-B, line 544 should reference Figure 8C, line 558 should reference Figure 8D, line 560 should reference Figure 8E, line 562 should reference Figure 8F-G and line 564 should reference Figure 8H.
    Response. *We have implemented the changes and ensured that all figures are now correctly referenced.*

****

Reviewer 3
Concise summary. *The authors satisfactory addressed my previous comments.*
Response*. We are pleased that our efforts have satisfactorily addressed reviewers' comments.*
Major issues. *None*
Minor issues. *None*

****

Reviewer 4
Concise summary. The authors are bringing the light on the immune cells present in the CNS with focusing on the characterization of the CD4 + T cells. By using the NHP model and cutting-edge assays, the authors are reporting an extensive phenotypic and transcriptomic of the CD4+ T Cells which is important information for the field. The authors edited their first version of the manuscript in a relevant way by answering reviewers' comments, concerns and by stating the limitation of their study. This manuscript will be informative for all people studying SIV/HIV infection in the CNS. ***Response***. We thank the reviewer for the recognition of our work.
Major issues. *None;* ***Minor issues.*** *None*
